# Neutrosophic statistical analysis of resistance depending on the temperature variance of conducting material

**DOI:** 10.1038/s41598-021-03347-z

**Published:** 2021-12-14

**Authors:** Usama Afzal, Hleil Alrweili, Naveed Ahamd, Muhammad Aslam

**Affiliations:** 1 Department of Physics, University of Education, Township Campus, Lahore, 54000 Pakistan; 2 Department of Mathematics, Faculty of Art and Science, Northern Border University, Rafha, Saudi Arabia; 3 Departments of Statistics, Faculty of Science, King Abdulaziz University, Jeddah, 21551 Saudi Arabia

**Keywords:** Mathematics and computing, Physics

## Abstract

In this work, we have proposed a neutrosophic statistical approach for the analysis of resistance of conducting material depending on the temperature variance. We have developed a neutrosophic formula and applied it to the resistance data. We also use the classical statistical approach for making a comparison between both approaches. As a result, it is observed that the neutrosophic statistical approach is more flexible and informative. Also, this work suggests that the neutrosophic statistical approach analyzes the resistance of conducting material for big data.

## Introduction

The electric properties of conducting material are studied mostly by analyzing the resistance variation. Generally, it is seen that the change in resistance variation of conducting material depends on temperature variation i.e. with an increase in the temperature ‘$\Delta T$’ the resistance of the conducting material ‘$\Delta R$’also increase^1^.

1 $$\Delta R \propto \Delta T$$

Because with an increase in temperature the vibrational motion of the atoms and molecules of conducting material increases, which becomes the reason for the electronic and atomic collision. As a result, the resistance of the conducting material increases. A number of researchers have worked on the resistance w.r.t temperature of different materials like graphene, carbon, and others, as can be seen in the following references^2–6^.

The resistance of the material can be measured through numerous methods^7,8^ as well as devices and data may be fixed-point values (determined values) or interval point values (undetermined values). This measured data can analyze through different statistical approaches. Generally, for fix point values i.e. when data has exact observation and imprecise, the classical statistical approach has been used^9^. For the interval point values i.e. having uncertainty, we cannot apply this approach. Fuzzy statistical logic was proposed by^10^, which deals with uncertain data^10^.

Smarandache introduced a reliable statistical approach is known as the neutrosophic statistical approach^11^, which is a more efficient and generalized form of the fuzzy statistical approach. Recently, neutrosophic approach use has been increased for the analysis of the interval data in a number of fields i.e. in applied sciences^12^, in medicine for diagnoses data measurement^13^, in astrophysics for measurement of earth speed data^14^ and in humanistic^15^. Reference^16^ used the K-means of the neutrosophic approach to analyze the data of the earthquake. Neutrosophic sampling was used by^17^ to study the limitation of health care for diagnosis. Similarly, the neutrosophic Kruskal–Wallis H test was used to analyze the data of COVID-19 by^18^. More information on the applications of neutrosophic logic in various fields can be seen in^17,19–21^.

The neutrosophic approach is used for the analysis of interval point value data i.e. having indeterminacy^9^ and has substantial benefit over the classical approach; because the classical statistic approach only deals with fix point value data i.e. having no indeterminacy. Moreover, the neutrosophic technique is more helpful than the classical technique. For example, the neutrosophic binomial distribution explains acceptance, rejection as well as indeterminate probabilities but on the other hand, classical binomial distribution only explains acceptance and rejection probabilities^22^. Similarities for more examples see following references^23–25^. Different statistical techniques were developed under neutrosophic statistics by Muhammad Aslam^26,27^.

The data of resistance with respect to change in temperature is measured in intervals. In the existing method, an average value of each interval is computed and used to study the relationship between resistance and temperature. By exploring the literature and according to the best of our knowledge, there is no work on studying these variables using neutrosophic statistics. In this paper, we will introduce neutrosophic statistics to analyze the resistance with respect to change in temperature data. We will compare the performance of the proposed method with the existing method under classical statistics. We expect that the proposed method will be efficient, effective and adequate to be applied for resistance with respect to change in temperature data.

## Methodology

Let ${X}_{iN}$ is the neutrosophic numbers having ${X}_{iL}$ lower values and ${X}_{iU}$ higher values, so the neutrosophic formula for the *ith* interval:

2 $${X}_{iN} = {X}_{iL}+ {X}_{iU}{I}_{N} \left(i = 1, 2, 3\dots {n}_{N}\right)$$

Here, *I*_*N*_* ∈ *[*I*_*L*_, *I*_*U*_] & *X*_*N*_* ∈ *[*X*_*L*_, *X*_*U*_] is a random neutrosophic variable having size *n*_*N*_* ∈ *[*n*_*L*_, *n*_*U*_]*.* The variable *X*_*iN*_* ∈ *[*X*_*iL*_, *X*_*iU*_] has two parts: lower value *X*_*iL*_ a classical part, and upper-value *X*_*iU*_*I*_*N*_ an indeterminate part having indeterminacy interval *I*_*N*_* ∈ *[*I*_*L*_, *I*_*U*_].

Similarly, neutrosophic mean $\overline{X }$_*N*_ $\in$ [$\overline{X }$_*L*_, $\overline{X }$_*U*_] is defined as follows:

3 $${\overline{X} }_{N} = {\overline{X} }_{L} + {\overline{X} }_{U}{I}_{N}; {I}_{N}\in \left[{I}_{L}, {I}_{U}\right]$$

Here, $${\overline{X} }_{U}={\sum }_{i=1}^{nL}({X}_{iL} / {n}_{L})$$ and $${\overline{X} }_{L}={\sum }_{i=1}^{nU}({X}_{iU} / {n}_{U})$$

As we are studying the change in resistance of a conducting material w.r.t. variation in temperature, so we can write the resistance as the function of the temperature i.e. *R*(*T*)*.* Now, let *R*(*T*)_*N*_ is measured interval value of the resistance i.e. *R*(*T*)_*N*_* ∈ *[*R*(*T*)_*L*_, *R*(*T*)_*U*_], here, *R*(*T*)_*L*_ and *R*(*T*)_*L*_ are the lower and upper values of the interval, respectively. The neutrosophic formula for resistance of the conducting material is written as follows:

4 $${R(T)}_{N} = {R(T)}_{L}+ {R(T)}_{U}{I}_{N}; {I}_{N}\in \left[{I}_{L}, {I}_{U}\right]$$

From the above resistance formula *R*(*T*)_*N*_* ∈ *[*R*(*T*)_*L*_, *R*(*T*)_*U*_] is an extension under the classical. The equation is containing two parts i.e. *R*(*T*)_*L*_ determined & *R*(*T*)_*U*_*I*_*N*_ indeterminate part. Moreover, *I*_*N*_* ∈ *[*I*_*L*_, *I*_*U*_] is known as an indeterminacy interval. Also, the measured resistance interval *R*(*T*)_*N*_* ∈ *[*R*(*T*)_*L*_, *R*(*T*)_*U*_] can be reduced to the classical or determined part if we choose *I*_*L*_ = 0. For classical analysis we use the classical average formula as given below:

5 $${R(T)}_{i}=\left({L}_{i}+{U}_{i}\right)/2$$

### Experiment

**Figure 1:**
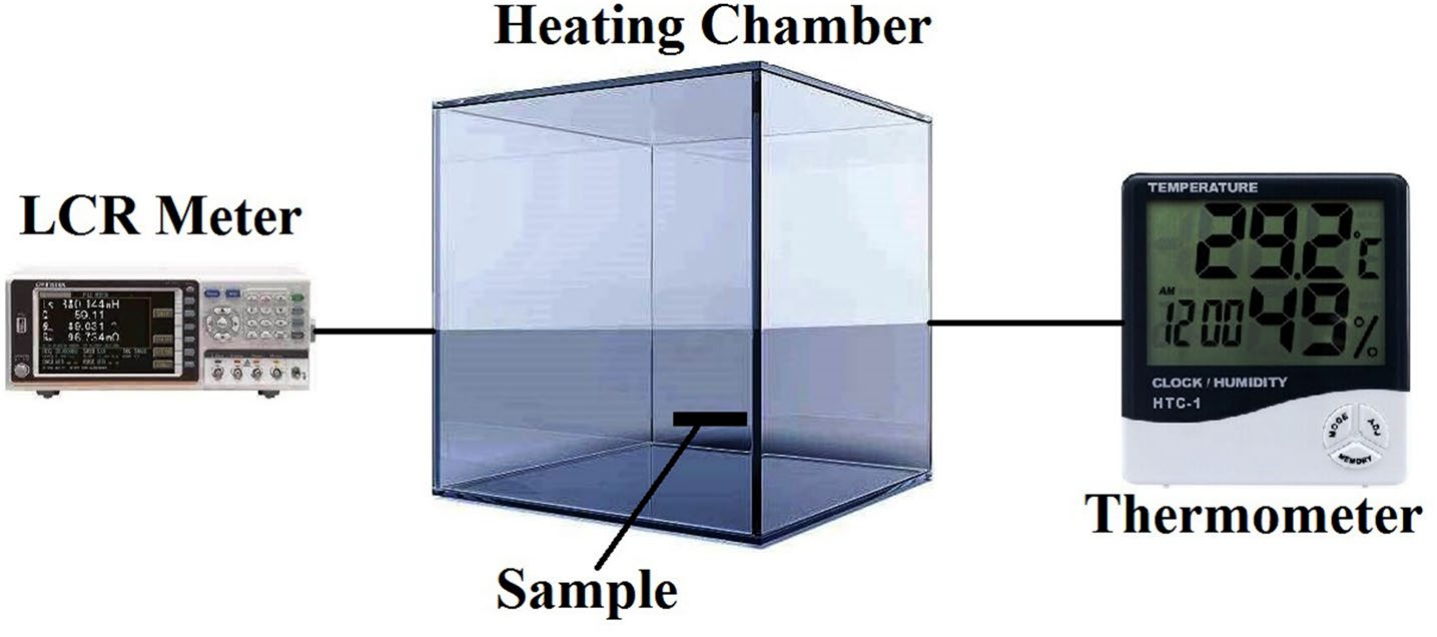
Characterization setup of measuring the resistance of a sample.

The main purpose of this research work is to express the advantages of neutrosophic a statistical approach for the analysis of measured resistance data with respect to (w.r.t) temperature variation of the conducting material. For this purpose, we have used a sample 200 nm thin film of a conducting material whose resistance values are measured at 100 Hz w.r.t. to change temperature through the LCR meter in intervals. The characterization setup is as shown in Fig. 1.

The measurement of the values has been performed in intervals i.e. at specific temperature value the maximum and minimum value change of resistance [min; max]. As we measured values in the interval, so for classical analysis, we convert these values into fix point. For using the neutrosophic approach, we first make a neutrosophic formula for resistance and then use this interval data for analysis.

## Results and discussion

The measured resistance values of the sample w.r.t change in temperature from 0 to 350 K (these values are not measured from experiment but by concerning the previous work on the resistance w.r.t temperature as in references^28,29^) as shown in Table 1.

**Table 1:** Resistance values with respect to temperature variation.

Temperature (K)	Resistance (Ω)
0	[0.120, 0.143]
25	[0.139, 0.167]
50	[0.157, 0.187]
75	[0.186, 0.215]
100	[0.212, 0.244]
125	[0.240, 0.273]
150	[0.266, 0.297]
175	[0.291, 0.326]
200	[0.317, 0.349]
225	[0.342, 0.375]
250	[0.369, 0.399]
275	[0.394, 0.422]
300	[0.421, 0.447]
325	[0.441, 0.466]
350	[0.461, 0.494]

### Analysis of data through neutrosophic and classical approach:

After measuring these values, we applied the neutrosophic method. For example, we have observed the first interval value of resistance [0.120; 0.143] at 0 K temperatures. Here *R*(*T*)_*L*_ is 0.120 and *R*(*T*)_*U*_ is 0.143. Similarly, indeterminacy *I*_*L*_ = 0 as reason has been mentioned above and *I*_*U*_ can find with formula *I*_*U*_ = (*R*(*T*)_*U*_ − *R*(*T*)_*L*_)*/R*(*T*)_*U*_. So, the value of *I*_*U*_ at a given resistance interval is 0.161. The neutrosophic form for the above capacitance interval is written as:

6 $${R(0K)}_{N} = 0.120+ 0.143{I}_{N}; {I}_{N}\in \left[0, 0.161\right]$$

For equation, one can select *I*_*N*_ = *0* for getting minimum value i.e. 0.120 or *I*_*N*_ = *0.161* for maximum value i.e. 0.143 and if choose any other value between *[0, 0.161]* for getting a value between the maximum and minimum value. Similarly, we can apply the neutrosophic statistics approach on all other the measured values of resistance w.r.t temperature changing from 0 to 350 K. For example, we have observed the value of resistance [0.120; 0.143] at 0 K temperatures. As it is in interval form, so we convert it into fixed point value i.e. 0.1315 by using the existing classical method.

### Comparison between classical statistical approach and neutrosophic statistical approach

In this section, we will compare the efficiency of the proposed method over the existing classical method in terms of a measure of indeterminacy, information and flexibility. The neutrosophic data and data using the classical method are shown in Table 2. The analysis using the resistance changing with respect to temperature variance data are shown in Table 2.

**Table 2:** Classical analysis of the resistance variance.

Temperature (K)	Resistance (Ω)		
	Neutrosophic analysis		Classical analysis
	Neutrosophic form	Indeterminacy	
0	R(0) ∈ 0.120 + 0.143I_N_	I_N_ ∈ [0, 0.023]	R(0) ∈ 0.132
25	R(25) ∈ 0.139 + 0.167I_N_	I_N_ ∈ [0, 0.167]	R(25) ∈ 0.153
50	R(50) ∈ 0.157 + 0.187I_N_	I_N_ ∈ [0, 0.161]	R(50) ∈ 0.172
75	R(75) ∈ 0.186 + 0.215I_N_	I_N_ ∈ [0, 0.135]	R(75) ∈ 0.201
100	R(100) ∈ 0.212 + 0.244I_N_	I_N_ ∈ [0, 0.131]	R(100) ∈ 0.228
125	R(125) ∈ 0.240 + 0.273I_N_	I_N_ ∈ [0, 0.121]	R(125) ∈ 0.257
150	R(150) ∈ 0.266 + 0.297I_N_	I_N_ ∈ [0, 0.104]	R(150) ∈ 0.282
175	R(175) ∈ 0.291 + 0.326I_N_	I_N_ ∈ [0, 0.107]	R(175) ∈ 0.307
200	R(200) ∈ 0.317 + 0.349I_N_	I_N_ ∈ [0, 0.092]	R(200) ∈ 0.333
225	R(225) ∈ 0.342 + 0.375I_N_	I_N_ ∈ [0, 0.088]	R(225) ∈ 0.359
250	R(250) ∈ 0.369 + 0.399I_N_	I_N_ ∈ [0, 0.075]	R(250) ∈ 0.384
275	R(275) ∈ 0.394 + 0.422I_N_	I_N_ ∈ [0, 0.066]	R(275) ∈ 0.408
300	R(300) ∈ 0.421 + 0.447I_N_	I_N_ ∈ [0, 0.058]	R(300) ∈ 0.434
325	R(325) ∈ 0.441 + 0.466I_N_	I_N_ ∈ [0, 0.054]	R(325) ∈ 0.453
350	R(350) ∈ 0.461 + 0.494I_N_	I_N_ ∈ [0, 0.067]	R(350) ∈ 0.478

From Table 2, it can be seen that in the classical method a fixed value is calculated on the basis of the average of each interval. Therefore, the classical analysis provides only one value at the time against a specific temperature for the conducting material. The classical method may mislead the decision-makers when extreme values are present in the intervals. On the other hand, the neutrosophic analysis used imprecise values and give information about the measure of indeterminacy. For example, when the temperature is 0 K, the classical analysis gives the value R(0) = 0.132. On the other hand, the proposed method provides the neutrosophic form R(0) = 0.120 + 0.143I_N_. According to the proposed method, the values of R(0) will be between 0.12 to 0.143 with measure of indeterminacy that is 0.023. By comparing both methods, it can be concluded that the existing method in Physics gives only information about the average value of the interval. On the other hand, the proposed method is quite flexible and adequate to be applied for the interval data.

**Figure 2:**
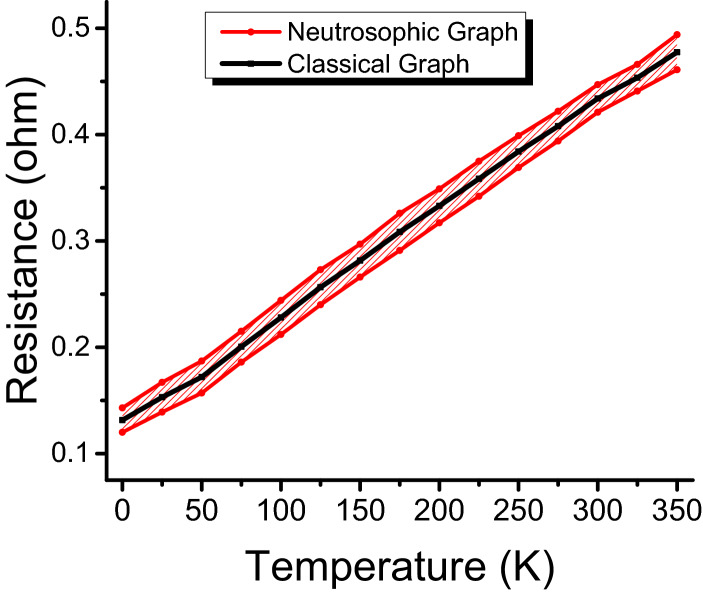
Classical statistical approach graph of resistance vs. temperature.

Now, we compare the proposed method with the classical method graphically. Let us draw the graphs for classical and neutrosophic analysis as shown in Fig. 2. From these graphs, it can be seen that graph of classical analysis is not much flexible because this graph is drawn at fix point values i.e. there is no indeterminacy. The graph using the proposed method is also shown in Fig. 2 which shows more flexibility. This means that the proposed method is more effective to analyze the resistance of the conducting material. From the graph, the information about the resistance at a specific value can be obtained with additional information about the measure of indeterminacy. Also, Fig. 2 shows that the neutrosophic approach is a generalization of the classical method as the curve using the classical method lies between the curves of the proposed method. As a result, it is found that neutrosophic statistics is informative, flexible and adequate than classical statistics.

## Conclusions

In this paper, the electrical property of the conducting material through resistance containing indeterminacy i.e. interval values have been analyzed. The neutrosophic statistical approach is used to analyze the interval values and the classical statistical approach is used to analyze the fix point values. From this study, it can be concluded that the analysis of resistance with respect to temperature of conducting material through the neutrosophic statistics approach is more informative, flexible and adequate than the analysis through classical statistics. Moreover, the proposed analysis suggests the use of a neutrosophic statistical approach for big data analysis as part of future research.

## Data Availability

The data is given in the paper.
